# Water Stress Responses of Tomato Mutants Impaired in Hormone Biosynthesis Reveal Abscisic Acid, Jasmonic Acid and Salicylic Acid Interactions

**DOI:** 10.3389/fpls.2015.00997

**Published:** 2015-11-18

**Authors:** Valeria A. Muñoz-Espinoza, María F. López-Climent, José A. Casaretto, Aurelio Gómez-Cadenas

**Affiliations:** ^1^Departament de Ciències Agràries i del Medi Natural, Universitat Jaume ICastelló de la Plana, Spain; ^2^Department of Molecular and Cellular Biology, University of GuelphGuelph, ON, Canada

**Keywords:** drought, *flacca*, hormonal crosstalk, *Solanum Lycopersicum*, *spr2*

## Abstract

To investigate the putative crosstalk between JA and ABA in *Solanum lycopersicum* plants in response to drought, *suppressor of prosystemin-mediated responses2* (*spr2*, JA-deficient) and *flacca (flc*, ABA-deficient) mutants together with the *naphthalene/salicylate hydroxylase (NahG)* transgenic (SA-deficient) line were used. Hormone profiling and gene expression of key enzymes in ABA, JA and SA biosynthesis were analyzed during early stages of drought. ABA accumulation was comparable in *spr2* and wild type (WT) plants whereas expression of 9-cis-epoxycarotenoid dioxygenase 1 *(NCED1)* and *NCED2* was different, implying a compensation mechanism between *NCED* genes and an organ-specific regulation of *NCED1* expression. JA levels and 12-oxo-phytodienoic acid reductase 3 (*OPR3*) expression in *flc* plants suggest that ABA regulates the induction of the *OPR3* gene in roots. By contrast, ABA treatment to *flc* plants leads to a reduction of JA and SA contents. Furthermore, different pattern of SA accumulation (and expression of isochorismate synthase and phenylalanine ammonia lyase 1) was observed between WT seedlings and mutants, suggesting that SA plays an important role on the early response of tomato plants to drought and also that JA and ABA modulate its biosynthesis. Finally, hormone profiling in *spr2* and *NahG* plants indicate a crosstalk between JA and SA that could enhance tolerance of tomato to water stress.

## Introduction

Drought is by far the most important environmental constrain in agriculture and every year the number of economic losses due to water constraints increases (Vicente-Serrano, 2007). This problem has been aggravated in the last decade by global climate changes.

Plants have developed a range of strategies to reduce the negative effects of drought on their physiology (Fang and Xiong, 2015). A complex network of highly coordinated hormonal interactions seems to be crucial for this process (Smekalova et al., 2014). Classically, the role of phytohormones have been described considering individual signaling pathways but this approach does not address the spatiotemporal specificity, considered central to fine-tune hormone signaling. Thus, the study of hormone profiling during early stages of water stress conditions in different mutant backgrounds could help to distinguish common from specific responses.

Perception of stress signals often causes changes in levels of different hormones to adapt and respond to environmental challenges (Shakirova et al., 2003; De Ollas et al., 2013, 2015; Rodríguez-Álvarez et al., 2015). Among phytohormones, abscisic acid (ABA), jasmonic acid (JA) and salicylic acid (SA) are known to play important roles in plant responses to stress.

Classically, ABA has been mainly related to abiotic stress (Gómez-Cadenas et al., 2002; Fujita et al., 2011). Structurally, ABA is a sesquiterpene derived from C_40_ oxygenated carotenoids with a number of steps controlling its synthesis. One of the key steps includes the cleavage of 9-cis-violaxanthin and/or 9-cis-neoxanthin to produce xanthoxin (North et al., 2007), a reaction catalyzed by 9-cis-epoxycarotenoid dioxygenase (NCED). Another important step is the oxidation of abscisic aldehyde by an abscisic aldehyde oxidase (AAO) which needs a molybdenum cofactor (MoCo) for its activity (Taylor et al., 1988).

By contrast, JA and SA have been considered signals of biotic stress threats for years because they fulfill essential roles in plant defense (Li et al., 2003). JA derives from linolenic acid via the octadecanoid pathway. There are several enzymes involved in this pathway, including lipoxygenase (LOX), allene oxide synthase (AOS), allene oxide cyclase (AOC) and 12-oxo-phytodienoic acid reductase (OPR). Although the function of these enzymes in JA biosynthesis under biotic stress situations is well understood (reviewed in Wasternack and Hause, 2013), only recent information is found on their role under drought. Indeed, research on the molecular mechanisms underlying hormonal regulation in response to drought has uncovered a complex and dynamic regulatory network in which JA and ABA participate (Harb et al., 2010; Brossa et al., 2011; Savchenko et al., 2014).

It is well-known that SA is involved in a broad range of physiological and metabolic responses in plants (Hayat et al., 2010). This phenolic compound can be synthesized from the primary metabolite chorismate via two distinct enzymatic pathways, one involving phenylalanine ammonia lyase (PAL) and the other isochorismate synthase (ICS; reviewed in Chen et al., 2009). Szepesi et al. (2009) claimed that osmotic adaptation in tomato depends on SA concentration and also highlighted the difficulties in experimentally recreating the fine-balance and timing for the endogenous levels of the hormone. For instance, when applied at low concentrations, SA and JA acted synergistically, whereas a high concentration of one hormone antagonized the other one (Mur et al., 2006).

Hormonal deficient mutants are an interesting model system to study hormonal interactions. In tomato, ABA-deficient mutants such as *sitiens* (Taylor et al., 1988), *flacca* (*flc*, Sagi et al., 1999) and *notabilis* (Burbidge et al., 1999) and JA-deficient genotypes such as *defenless1* and *suppressor of prosystemin-mediated responses2* (*spr2*, Li et al., 2003) have been described. *Spr2* plants are impaired in a fatty acid desaturase required for JA biosynthesis whereas *flc* plants have limited ABA biosynthesis due to impairment in a MoCo sulfurase, a cofactor needed for AAO function. Salicylic acid-deficient *NahG* (naphthalene/salicylate hydroxylase) transgenic line has been also used (Jia et al., 2013). It overexpresses the *NahG* gen from *Pseudomonas putida* that degrades SA to catechol, showing low levels of this hormone. Previous research with these mutants indicated an enhanced basal resistance to biotic factors mediated by SA (Audenaert et al., 2002; Avila et al., 2012), which suggest that SA can also interact with ABA and JA in the physiological responses to stress conditions (De Torres Zabala et al., 2009).

The research presented in this paper was designed to better understand the crosstalk between JA, SA, and ABA in tomato plants in response to drought. For this purpose, different genotypes with impaired production of hormones were used. Data indicate that not only JA and ABA are part of the signaling network controlling the responses of tomato plants to water stress conditions, but also SA is relevant.

## Materials and methods

### Plant material

Seeds of *Solanum lycopersicum* L. were germinated in trays with vermiculite and after 1 month, they were transplanted to 2.5 L pots filled with perlite as a substrate. The following genotypes were used: *spr2, flc, NahG* and the respective wild-type (WT) cultivars: Castlemar (CSL), Ailsa Craig (AC), and Moneymaker (MM). Plants were watered three times a week with 0.5 L of a half-strength Hoagland solution and allowed to acclimate for 3 months in a greenhouse with a 16 h photoperiod (maximum PAR 1200 μm m^−2^ s^−1^), 26 ± 4 °C day temperature, 18 ± 3 °C night temperature, and relative humidity ranging from 60 to 90%. Four-month-old plants were used for all the experiments.

### Water stress conditions

After the acclimation period, 20 plants of each genotype (AC, CSL, MM, *spr2, flc, and NahG*) were randomly distributed. Drought stress was imposed by transplanting plants to dry perlite (controls were transplanted to wet perlite at the same time). Within each group, leaves (from an intermediate position in the shoot) and young roots from four plants were collected at 0, 1, 3, 6, and 24 h after imposing the stress conditions. Tissue was immediately frozen in liquid nitrogen and stored at −80°C until further analyses. For each of analyses described below (plant hormone profiling and RNA extraction), three independent extractions per group were performed.

### ABA application

A different group of 32 *flc* plants was used for ABA treatment. Half of the plants, randomly distributed, was treated with a 10 μM ABA solution (Sigma Aldrich, Madrid, Spain) whereas the other was watered with a mock solution as in (De Ollas et al., 2013). Preliminary experiments indicated that this ABA concentration had an effect on gene expression without having toxic effects on plants (data not shown). After 1 week of treatment, water stress was applied in both groups of plants as described above. Plants were collected at 0, 3, 6, and 24 h after initiating drought stress.

### Leaf relative water content

Plant water status was determined at each time point by measuring the relative water content (RWC) (Turner, 1981). After sampling, leaf fresh weights (FW) were determined, and then leaves were hydrated with distilled water for 24 h at ambient temperature in darkness until saturation. The leaves were then reweighed to obtained leaf turgid weights (TW). Subsequently, leaves were oven dried at 60°C for 3 days and their dry weights determined (DW). Leaf RWC was calculated following the formula; RWC % = (FW − DW)/(TW − DW)^*^100.

### Chlorophyll fluorescence measurements

Quantum yield (ΦPSII) measurements were performed with an OS 1 FL portable fluorometer (Opti-Sciences, Tyngsboro, MA, USA) in 12 light-adapted leaves per plant. Four plants per genotype were randomly chosen as replicates (López-Climent et al., 2008).

### Hormone analysis

Hormone extraction and analysis were carried out following the procedure described in Durgbanshi et al. (2005) with slight modifications. Frozen fresh tissue (0, 2 g) was spiked with 100 ng of d_6_-ABA, 100 ng of dihydrojasmonic acid and 100 ng of d_6_-SA (prepared as in Gómez-Cadenas et al. (2002) and homogenized with 5 ml of distilled water. After centrifugation at 4000 × g at 4°C, supernatants were recovered and pH adjusted to 3 with 30% acetic acid. The acidified water extract was partitioned twice against 3 ml of diethyl ether. The organic upper layer was recovered and vacuum evaporated in a centrifuge concentrator (Speed Vac, Jouan, Saint HerblainCedex, France). The dry residue was then resuspended in a 10% MeOH solution by gentle sonication. The resulting solution was passed through 0.22 μm regenerated cellulose membrane syringe filters (Albet S.A., Barcelona, Spain) and directly injected into a UPLC system (Acquity SDS, Waters Corp., Milford, MA, USA).

Analytes were separated by reversed-phase (Nucleodur C18, 1.8 μm 50 × 2.0 mm, Macherey- Nagel, Barcelona, España) using a linear gradient of ultrapure H_2_O (A) and MeOH (B) (both supplemented with 0.01% acetic acid) at a flow rate of 300 μl min^−1^. The gradient used was: (0–2 min) 90:10 (A:B), (2–6 min) 10:90 (A:B), and (2–6–7 min) 90:10 (A:B). Hormones were quantified with a Quattro LC triple quadrupole mass spectrometer (Micromass, Manchester, UK) connected online to the output of the column through an orthogonal Z-spray electrospray ion source. Quantitation of plant hormones was achieved by external calibration with standards of known amount.

### RNA extraction and cDNA synthesis

Total RNA was extracted by using the E.Z.N.A. Kit (OMEGA Bio-Tek, Norcross, GA, USA) from frozen plant material according to the manufacturer's instructions. RNA integrity was visualized in 1% agarose gels while concentration and purity (260/280 ratio) was determined with a NanoDrop 2000 spectrophotometer (Thermo Scientific, Waltham, MA, USA). RNA was treated with DNase I (Fermentas, Hanover, MD, USA) to remove DNA contamination. First-strand cDNA was carried out using 2 μg total RNA with the PrimerScript RT-PCR kit from Takara (Condalab, Barcelona, Spain) following the manufacture's protocol.

### Real-time qRT-PCR analysis

Real-time qRT-PCR was performed using an ABI StepOne Detection System (Applied Biosystems, USA) and the Maxima SYBR Green/ROX qPCR mix from Fermentas (Thermo Scientific Fermentas, Spain). The primer details for the qRT-PCR are provided as supporting data (Table S1). Thermocycling conditions were: 95°C for 10 min followed by 40 cycles of 95°C for 10 s, 60°C for 10 s and 72°C for 20 s. Fluorescent intensity data were acquired during the extension time. The specificity of the reaction was verified by melting curve analysis and relative quantification of the expression was done using the Relative Expression Software Tool (REST, Pfaffl et al., 2002). Normalization was performed using the expression levels of the *GADPH* gene. Results are expressed as the average of three independent replicate ± SE.

### Statistical analysis

Statistical analyses were performed using SPSS-10 statistical software (SPSS Inc., Chicago, IL, USA). Differences between mutants and WT plants were compared by using the least significant difference (LSD) test (*P* ≤ 0.05).

## Results

### RWC and ΦPSII in *spr2* and *flc* plants

To investigate whether the *spr2* and *flc* mutations had an effect on signaling events in the early stages of water stress, both mutants were evaluated in comparison with WT, following a time-course experiment. Visible symptoms of leaf wilting were observed after stress imposition in WT and mutant plants. However, all plants recovered their turgid aspect when they were rehydrated after a 24 h-period of water stress (see Figure S1).

Differences in water content were evident after one h of stress in all groups of plants. However, the *flc* mutant was the most affected (Figure S2). RWC decreased throughout the experimental period in the four groups of plants. Stress conditions caused an important loss of leaf water in s*pr2* and CSL plants, and RWC followed a similar pattern in both genotypes (Figure S2A). At the end of experiment, the RWC reached the minimum value, 67.4 and 63.6%, in *spr2* and CSL, respectively. Compared to WT, *flc* plants lost 20% more water content after 6 h of stress (Figure S2B). This difference was similar after 24 h of water deprivation. The highest values of RWC were observed in AC, suggesting a better capacity to regulate water status during the stress condition.

Water stress reduced ΦPSII in both groups of plants (Figures S2C,D). In CSL and *spr2* plants, ΦPSII only decreased at the end of experiment, with a reduction of 17.6% in CSL and 7.1% in *spr2* with respect control plants. However, no differences were observed between CSL and *spr2*. In contrast, ΦPSII considerably decreased after 3 h of water deprivation in *flc* mutants showing remarkable differences with respect to WT plants although ΦPSII values were similar in mutant and WT plants at the end of experiment.

### Hormonal balance during early stages of water stress

**Figure 1:**
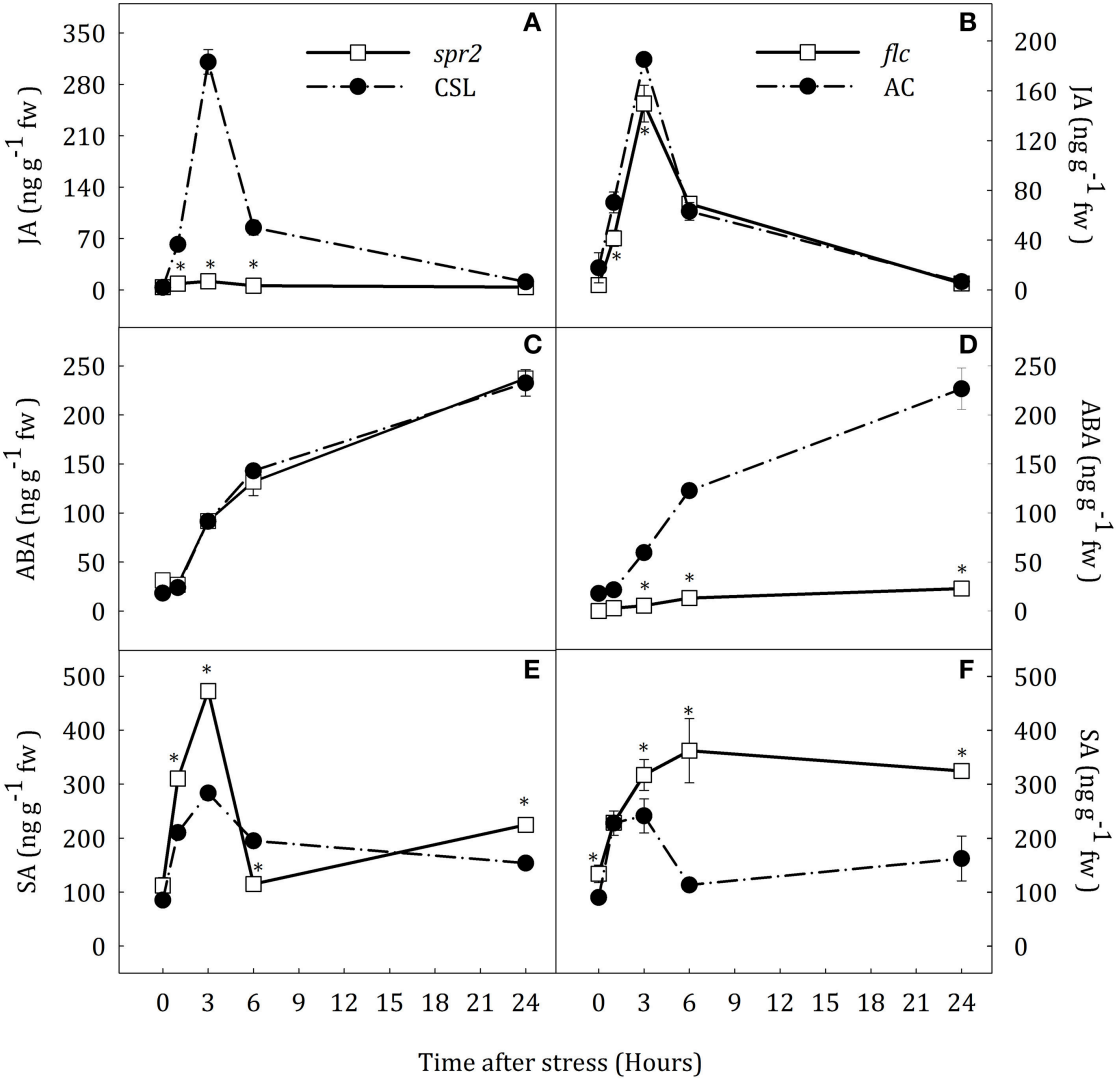
**JA (A,B), ABA (C,D) and SA (E,F) levels in roots of *Solanum lycopersicum* [WT (CSL and AC, black circles) and mutants (*spr2* and *flc*, white square)] under control (*t* = 0) and water-stress conditions**. Data are mean values ± standard deviation of three independent determinations. Asterisks denote statistical difference with respect to WT at *p* ≤ 0.05.

**Figure 2:**
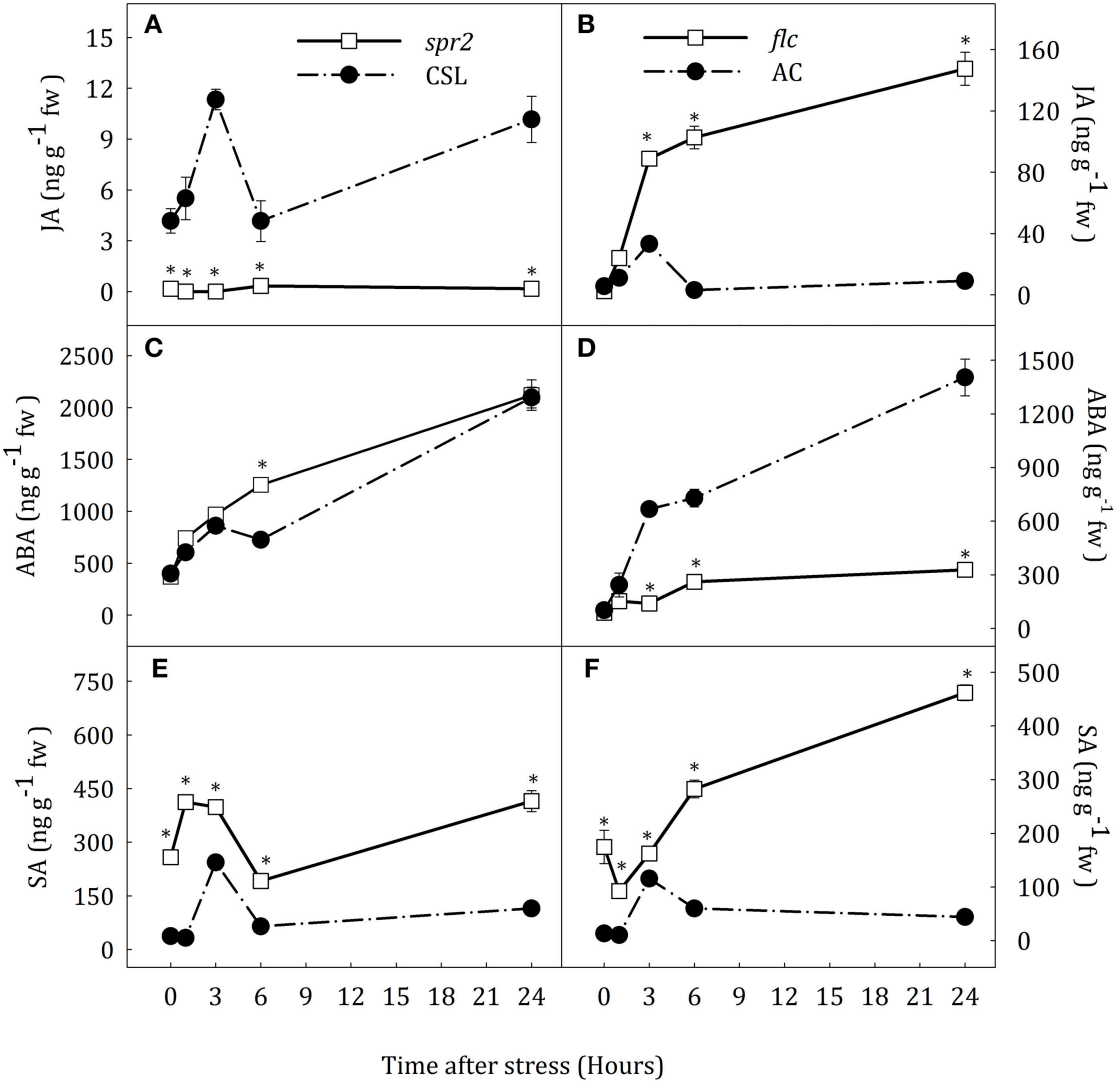
**JA (A,B), ABA (C,D) and SA (E,F) endogenous concentrations in leaves of *Solanum lycopersicum* [WT (CSL and AC, black circles) and mutants (*spr2* and *flc*, white square)] under control (*t* = 0) and water-stress conditions**. Data are mean values ± standard deviation of three independent determinations. Asterisks denote statistical difference with respect to WT at *p* ≤ 0.05.

JA, ABA, and SA levels were determined as indicators of the hormonal status in roots and leaves of *spr2* and *flc* mutants (Figures 1, 2).

#### Roots

Hormonal profiles were slightly different in roots of *spr2* and *flc* mutants but similar between their respective WT plants (Figure 1). Water stress induced a rapid increase of endogenous JA concentration which reached maximum values at 3 h after the stress imposition in CSL, AC and *flc* plants (Figures 1A,B). JA content in *flc* was significantly lower than in AC at 3 and 6 h. Interestingly, the same result was observed in similar experiments with *notabilis* mutants that have limited ABA biosynthesis due to a null mutation in the *NCED1* gene (see Supplementary Data Figure S3A). After this transient increase, root JA content in stressed plants decreased reaching basal levels. *Spr2* plants showed basal levels of JA throughout the experimental period (Figure 1A). The increase of ABA levels was progressive from 3 h in WT and *spr2* plants, reaching values 12.6-fold higher than in well-watered plants at the end of the experiment (Figures 1C,D). Compared to other species, the lack of transient JA accumulation did not affect the progressive accumulation of ABA. As expected, *flc* plants showed basal levels of ABA throughout the experimental period (Figure 1D). Water stress induced a transient increase in SA concentration in CSL and AC plants (3.3- and 2.7-fold higher than levels in well-watered plants at 3 h of stress; Figures 1E,F). In addition, *spr2* and *flc* mutants showed different levels of SA accumulation. The increase of SA content in *spr2* plants was 1.7-fold higher than in CSL plants. However, after 6 h of water stress, SA levels decreased to basal levels (Figure 1E). At the end of the experiment, SA levels in *spr2* roots were slightly higher than in CSL (1.4-fold). On the contrary, SA concentration in *flc* mutants progressively increased from the beginning of the experiment, reaching levels 2.4-fold higher than in well-watered plants at the end of experimental period (Figure 1F).

#### Leaves

Hormone profiles in leaves of CSL and AC plants were similar to those in roots. Water deprivation caused a transient increase in JA and SA concentration at 3 h of stress and a progressive increase of ABA content (Figure 2). In leaves of *spr2* plants, JA concentration was almost constant at basal levels. Again, ABA accumulation pattern in this JA-deficient mutant was similar to that in CSL (Figures 2A,C). In contrast, leaf JA content progressively increased in *flc* mutants, being 16.3-fold higher than in AC, at the end of the experiment. The endogenous concentration of ABA in *flc* plants was 76.6% lower than in AC seedlings at the end of experiment (Figures 2C,D). Similarly to that observed in roots, *flc* and *spr2* mutants showed a different pattern of SA accumulation. Leaf SA levels of both mutants were higher than in their respective WT backgrounds throughout the experiment (Figures 2E,F). *Spr2* plants showed a slight peak in SA content after the stress imposition, reaching maximum levels 3 h after the stress onset (1.6-fold increase with respect to the control levels). In *flc* mutants by contrast, SA levels progressively increased throughout the experimental period after a slight reduction. The highest concentration of SA was observed in *spr2* and *flc* plants at the end of the experiment (Figures 2E,F).

Our data indicate that JA deficiency do not affect ABA accumulation during water stress. Furthermore, the alteration of SA and JA concentrations in response to water stress suggests an involvement of these hormones in signaling this condition.

### Gene expression pattern for key genes of JA and ABA biosynthesis pathways

**Figure 3:**
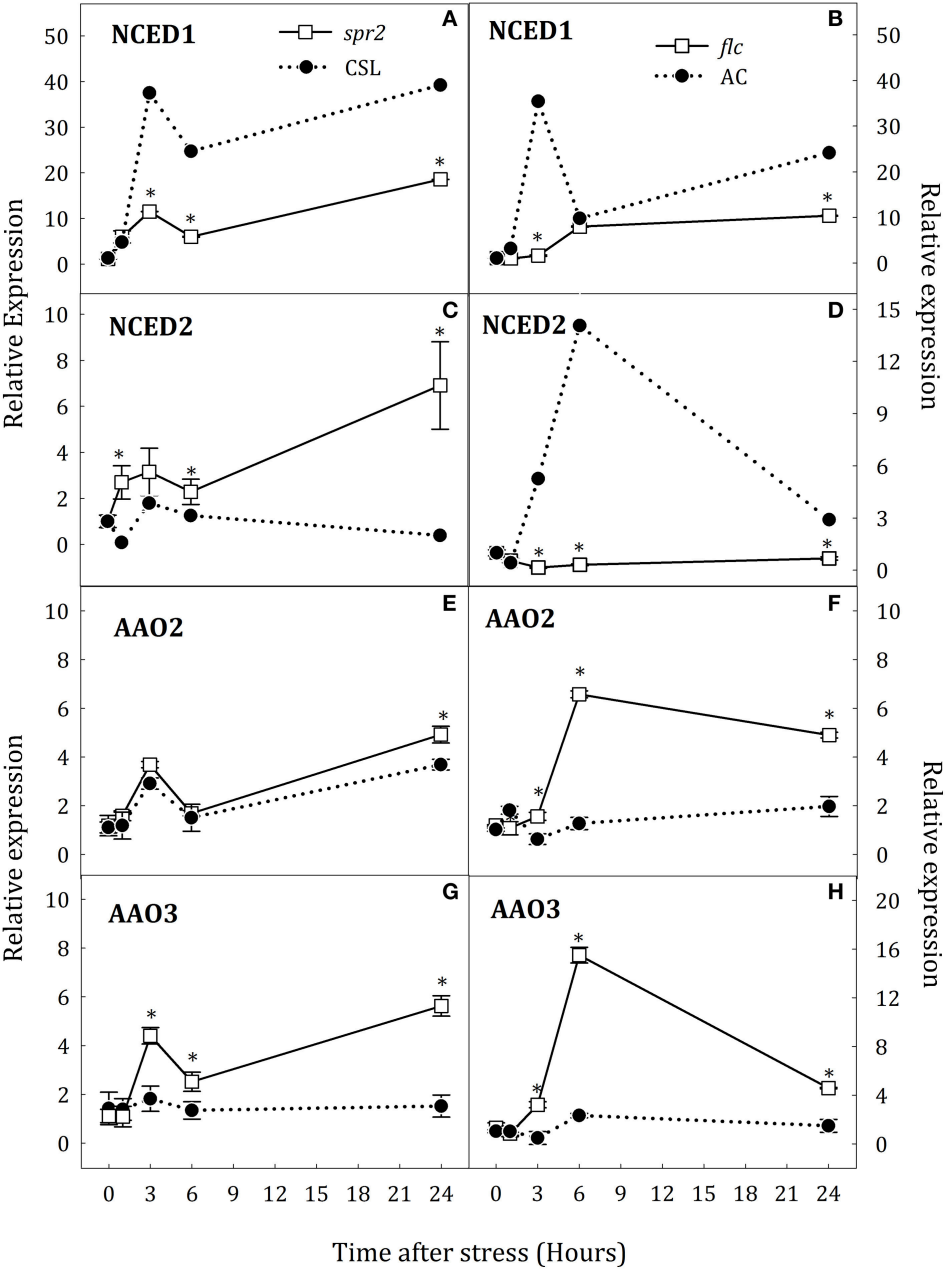
**Relative expression of *NCED1* (A,B), *NCED2* (C,D), *AAO2* (E,F), and *AAO3* (G,H) in roots of *Solanum lycopersicum* [WT (CSL and AC, black circles) and mutants (*spr2* and *flc*, white square)] under control (*t* = 0) and water-stress conditions**. Data are mean values ± standard deviation of three independent determinations. Asterisks denote statistical difference with respect to WT at *p* ≤ 0.05.

**Figure 4:**
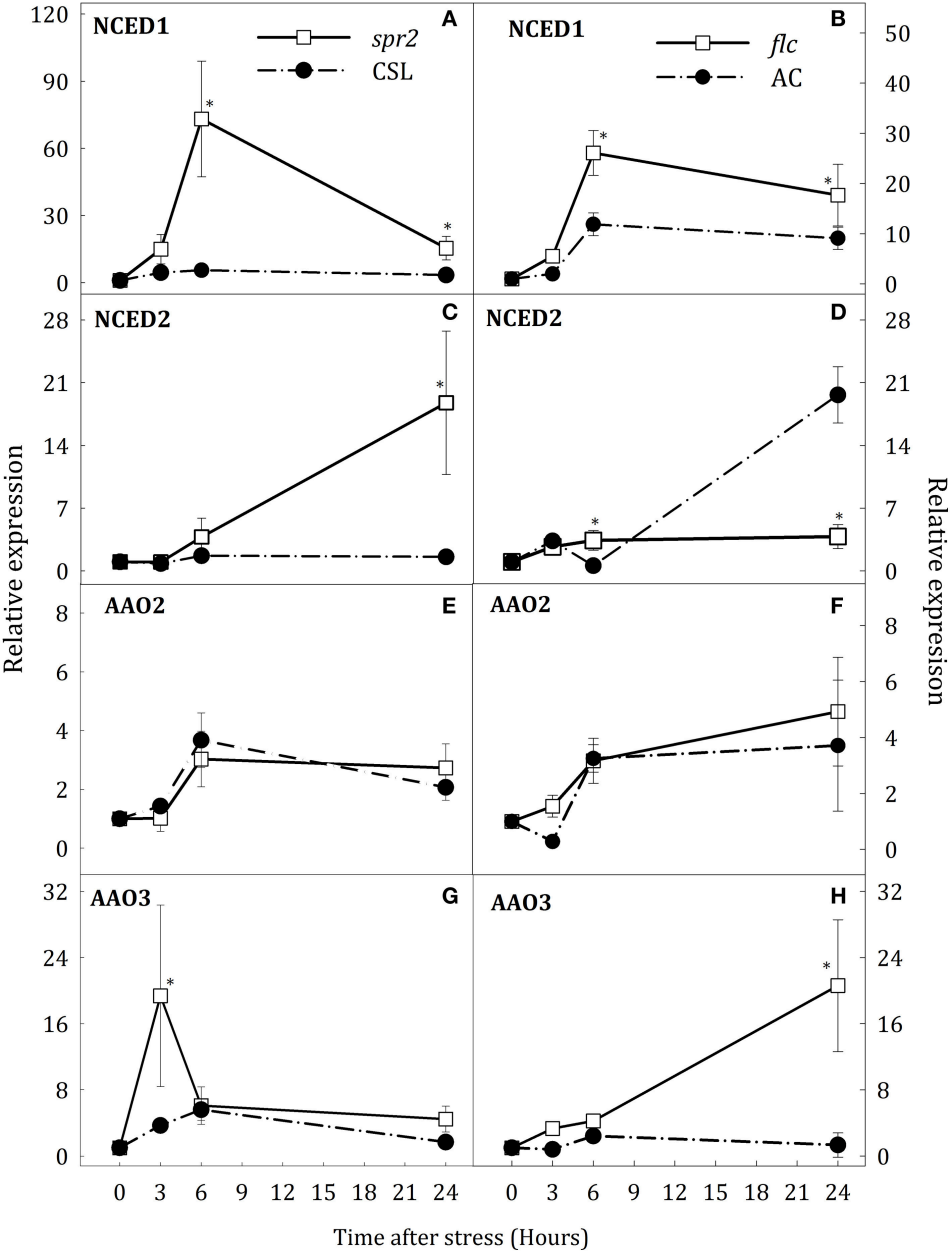
**Relative expression of *NCED1* (A,B), *NCED2* (C,D), *AAO2* (E,F), and *AAO3* (G,H) in leaves of *Solanum lycopersicum* [WT (CSL and AC, black circles) and mutants (*spr2* and *flc*, white square)] under control (*t* = 0) and water-stress conditions**. Data are mean values ± standard deviation of three independent determinations. Asterisks denote statistical difference with respect to WT at *p* ≤ 0.05.

To determine whether altered levels of JA and ABA during water stress could also affect the expression of key genes for the biosynthesis of both hormones, gene expression was evaluated in *spr2* and *flc* plants. Water deprivation strongly induced expression of *NCED1* and slightly that of *NCED2* in WT roots. *NCED1* transcript levels were clearly down-regulated in roots of both mutants with respect to WT (Figures 3A,B). In *spr2* plants, the increase of *NCED2* transcript levels was higher than in CSL plants (17.8-fold higher at 24 h, Figure 3C). By contrast, in *flc* plants, *NCED2* expression was not induced throughout the experimental period (Figure 3D). In leaves of stressed plants, *NCED1* was up-regulated in *spr2* and *flc* mutants after 6 h of stress (Figures 4A,B) whereas *NCED2* transcripts were only different in both mutants with respect to WT at 24 h of stress imposition (Figures 4C,D). Again, as in roots, the trend was different in both mutants: *NCED2* expression in *spr2* was higher than in CSL and in *flc* lower than in AC.

Transcript levels of the three *AAO* genes were also determined. In stressed roots, no changes in the expression of *AAO1* were observed and, therefore, data have been omitted. However, *AAO2* and *AAO3* were up-regulated in *spr2* and *flc* mutants (Figures 3E–H). In *spr2* plants, the increase of *AAO2* transcript levels was slightly higher than in WT but *AAO3* expression was significantly elevated (4.2-fold higher than in CSL plants at 24 h, Figures 3E,G). Similar trends were observed in *flc* mutants (Figures 3F,H), as *AAO2* and *AAO3* transcript levels were strongly induced after 6 h of stress (34.4 and 73.2% higher than WT, respectively), and remained high at the end of experiment. In leaves, water stress conditions induced the expression *AAO2* and *AAO3* but, contrary to roots, scarce differences were observed between WT plants and mutants (Figures 4E–H). Levels of *AAO2* transcripts were similar in mutants and WT as *AAO2* transcript abundance increased after 6 h of water stress (Figures 4E,F). However, *AAO3* expression was slightly higher in stressed *spr2* and *flc* plants than in WT (Figures 4G,H).

**Figure 5:**
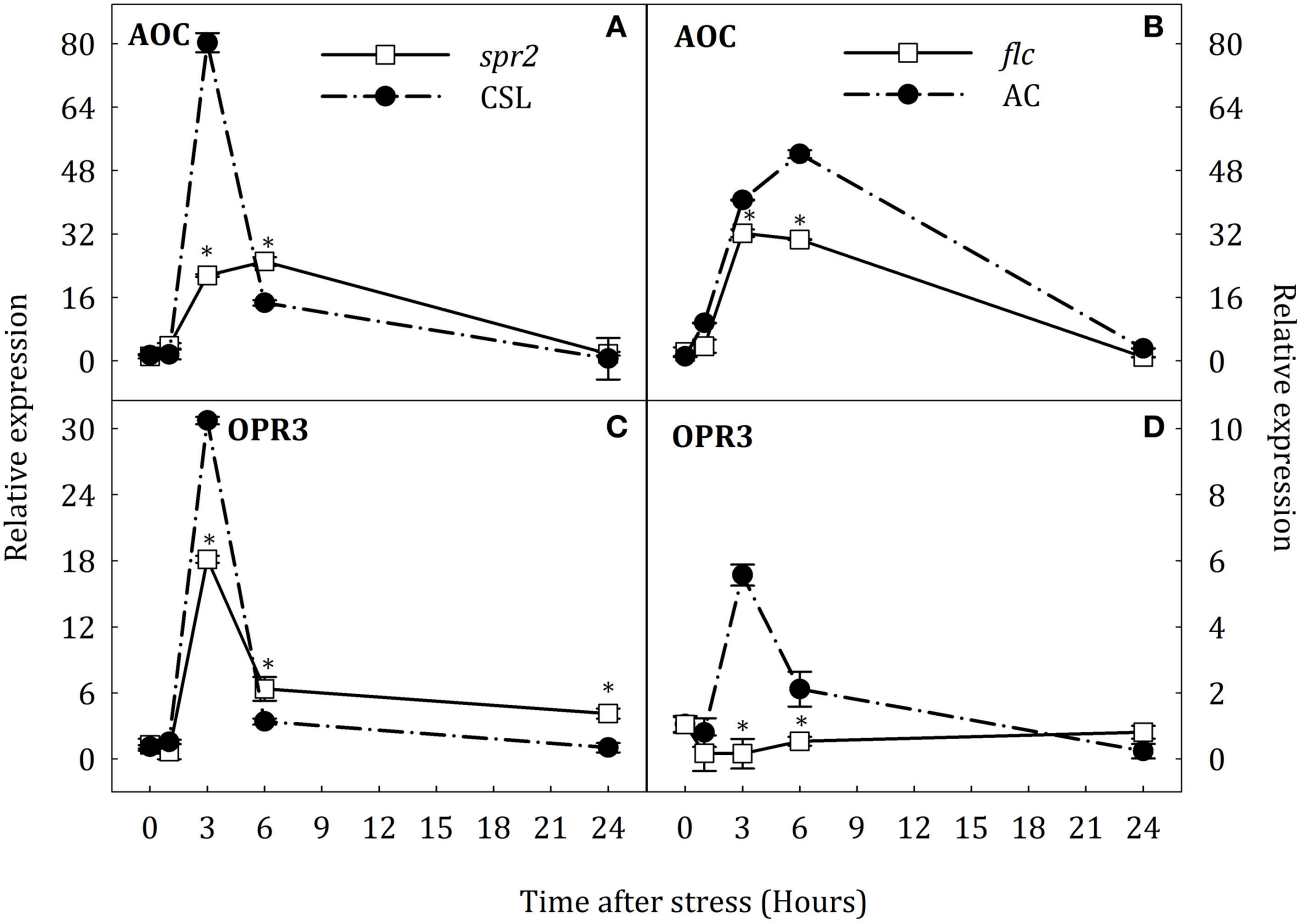
**Relative expression of *AOC* (A,B) and *OPR3* (C,D) in roots of *Solanum lycopersicum* [WT (CSL and AC, black circles) and mutants (*spr2* and *flc*, white square)] under control (*t* = 0) and water-stress conditions**. Data are mean values ± standard deviation of three independent determinations. Asterisks denote statistical difference with respect to WT at *p* ≤ 0.05.

**Figure 6:**
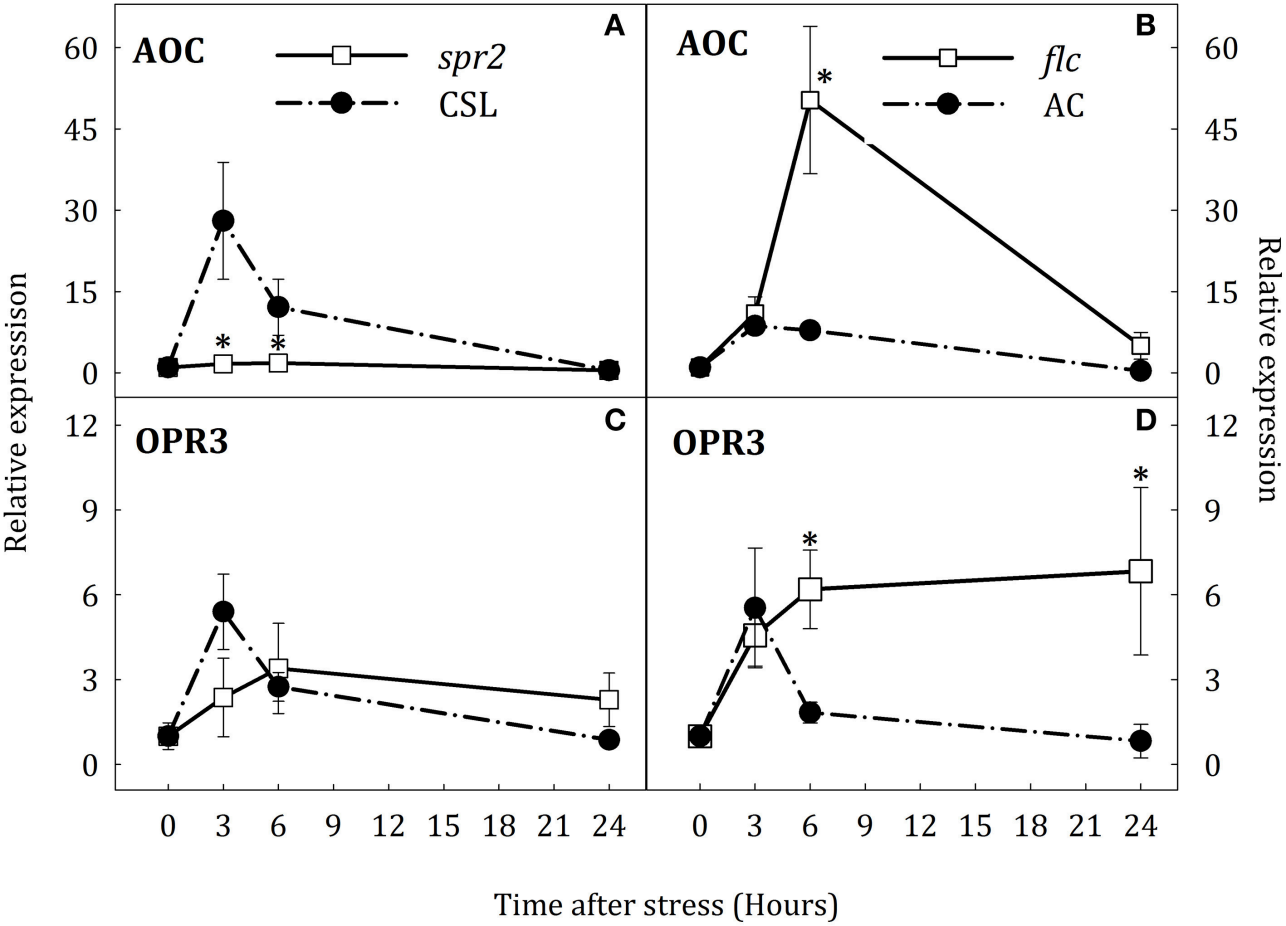
**Relative expression of *AOC* (A,B) and *OPR3* (C,D) in leaves of *Solanum lycopersicum* [WT (CSL and AC, black circles) and mutants (*spr2* and *flc*, white square)] under control (*t* = 0) and water-stress conditions**. Data are mean values ± standard deviation of three independent determinations. Asterisks denote statistical difference with respect to WT at *p* ≤ 0.05.

In response to stress, expression of *AOC* and *OPR3* was induced in roots and leaves of WT plants (Figures 5, 6). In roots, *AOC* expression in *spr2* and *flc* mutants was lower than in their respective WT genotypes after 3 h of stress (Figures 5A,B). *OPR3* expression was induced at the same time in CSL, AC, and *spr2* seedlings. In this case, *OPR3* expression was lower in *spr2* than in CSL (Figure 5C). Surprisingly, transcript levels of *OPR3* did not increase in *flc* mutants under the stress condition, remaining above basal levels throughout the experiment (Figure 5D). In contrast, no major changes in the expression of *AOC* were observed in leaves of *spr2* plants although the expression of this gene, as in roots, was strongly induced in CSL after 3 h of stress (Figure 6A). In addition, the expression profile of *OPR3* was similar in *spr2* and CSL plants, showing a slight increase after 3 h of stress and returning to basal levels at 24 h (Figure 6C). In *flc* and AC leaves, *AOC*, and *OPR3* expression did not decrease after 6 h of stress (Figures 6B,D).

These results suggest an organ-specific regulation of *NCED1* expression and compensative mechanisms to induce ABA accumulation in *spr2* mutant. Furthermore, data indicate that SA may participate in ABA biosynthesis by induction of *AAO3* expression. On the other hand, results obtained in *flc* mutants evidence that endogenous ABA levels may modulate *OPR3* expression in roots and leaves.

### Gene expression pattern for key genes in SA biosynthesis

**Figure 7:**
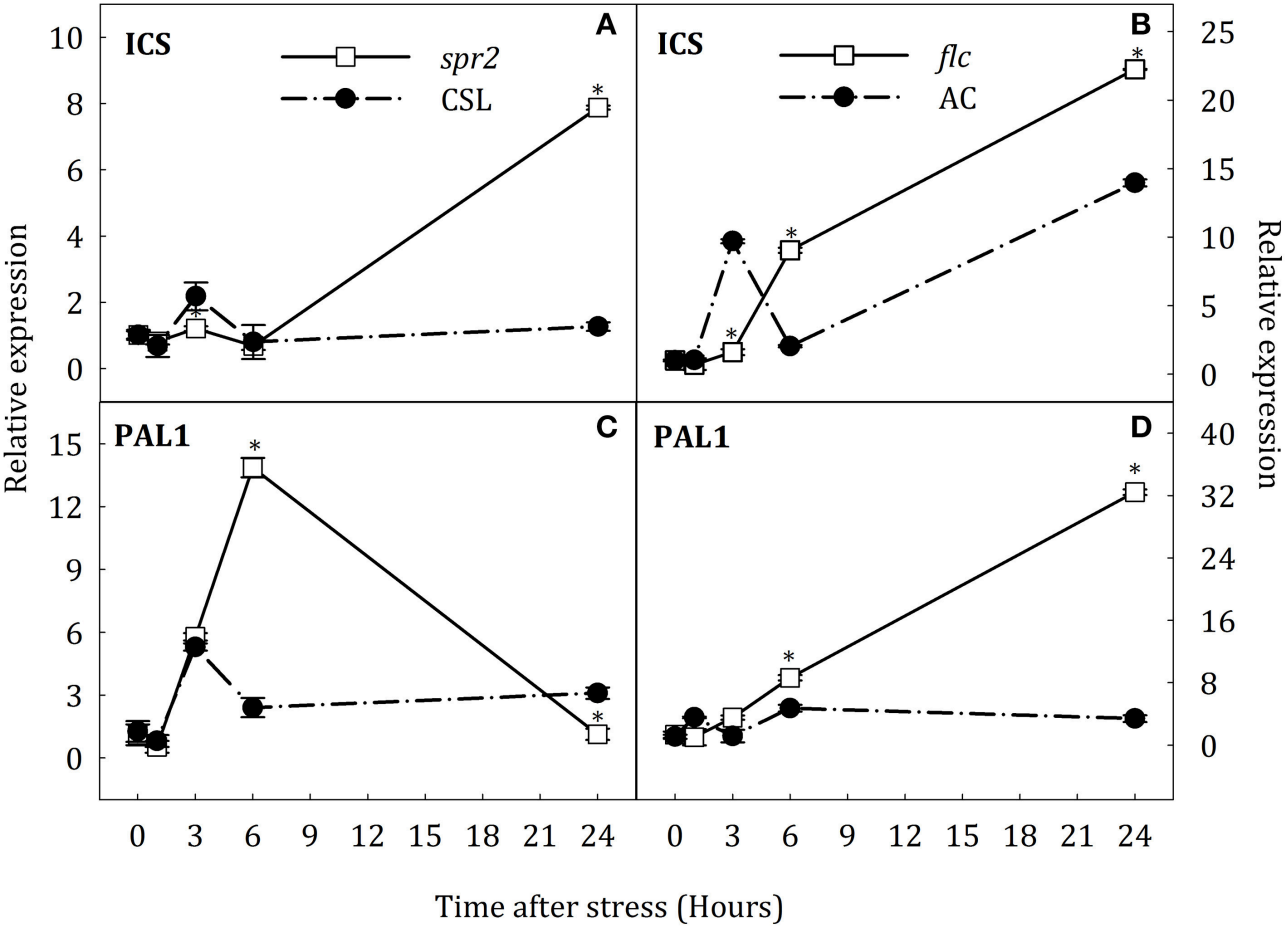
**Relative expression of *ICS* (A,B) and *PAL1* (C,D) in roots of *Solanum lycopersicum* [WT (CSL and AC, black circles) and mutants (*spr2* and *flc*, white square)] under control (*t* = 0) and water-stress conditions**. Data are mean values ± standard deviation of three independent determinations. Asterisks denote statistical difference with respect to WT at *p* ≤ 0.05.

**Figure 8:**
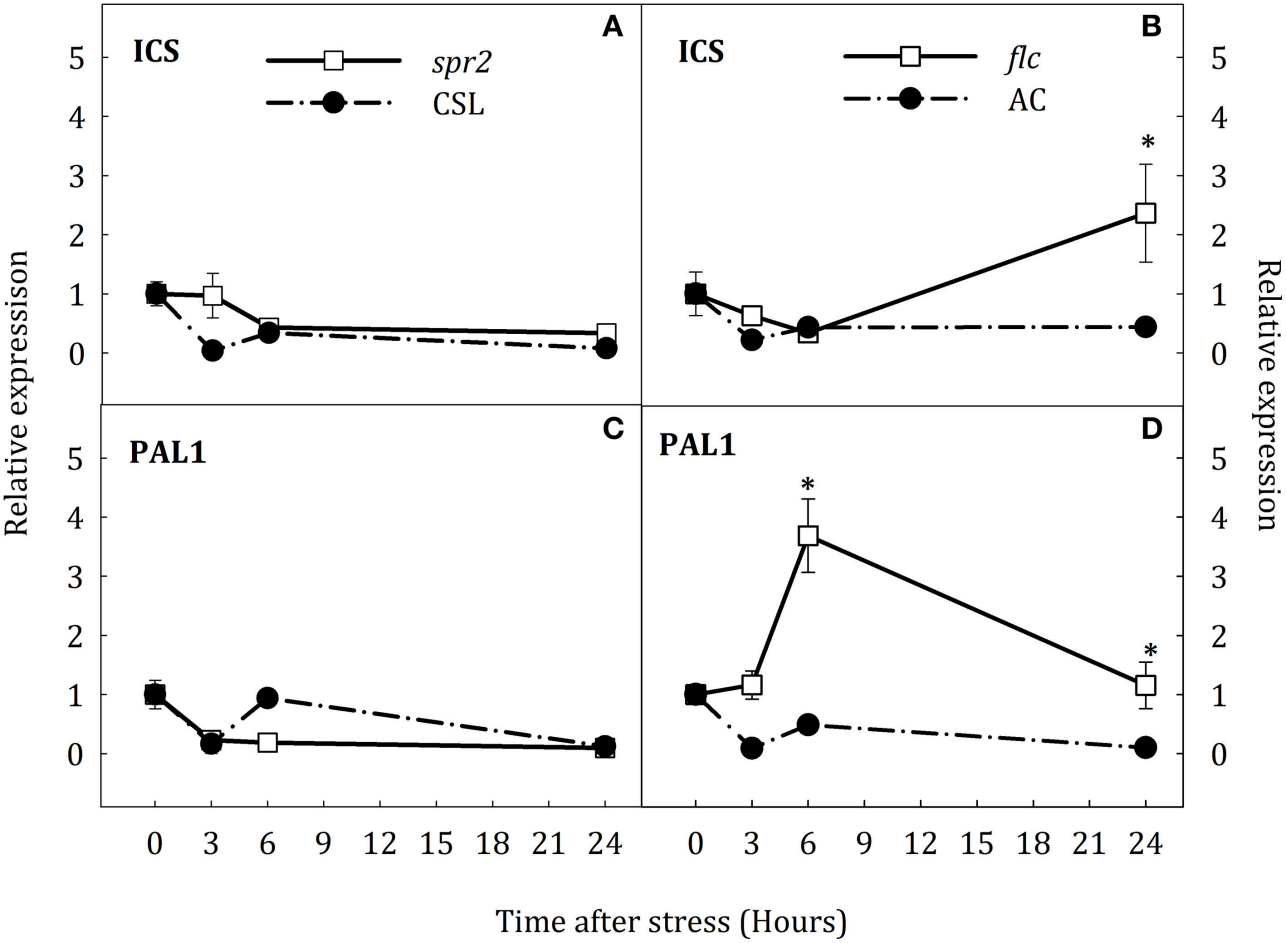
**Relative expression of *ICS* (A,B) and *PAL1* (C,D) in leaves of *Solanum lycopersicum* [WT (CSL and AC, black circles) and mutants (*spr2* and *flc*, white square)] under control (*t* = 0) and water-stress conditions**. Data are mean values ± standard deviation of three independent determinations. Asterisks denote statistical difference with respect to WT at *p* ≤ 0.05.

To test the hypothesis that SA accumulation under water stress could result from the novo biosynthesis of this hormone and that JA- and ABA- deficiency could affect the expression of key genes in the SA biosynthesis, the expression of *ICS* and *PAL1* was evaluated by qRT-PCR in roots and leaves of all groups of plants. The expression of both genes in roots showed a transient up-regulation at the early stages of stress in AC and CSL plants (Figure 7). In *spr2* mutants, *ICS* transcript levels considerably increased at the end of stress (8.2-fold higher than in CSL, Figure 7A). Moreover, *PAL1* expression in *spr2* mutants was twice as high as in CSL plants at 6 h of stress, reverting to control levels by the end of experiment (Figure 7C). In *flc* mutants, *ICS* and *PAL1* transcripts increased after 6 h of stress, remaining higher than in AC at 24 h (1.6-fold for *ICS* and 9.4-fold for *PAL1*, with respect to WT, Figures 7B,D). In stressed leaves, *ICS* and *PAL1* were down-regulated in *spr2* plants (Figures 8A,C). In contrast, *ICS* transcripts increased at the end of the experiment in *flc* mutants (2.5-fold higher than in well-watered plants, Figure 8B) and *PAL1* expression increased throughout the experimental period (Figure 8D). WT genotypes (CSL and AC) showed low levels of *ICS* and *PAL1* transcripts throughout the experiment. These results indicate that accumulation of SA could result, at least in part, from *de novo* synthesis, by induction of *ICS* and *PAL1* expression. They also point to an interaction of JA and ABA on SA biosynthesis.

### ABA treatment in *flc* plants

**Figure 9:**
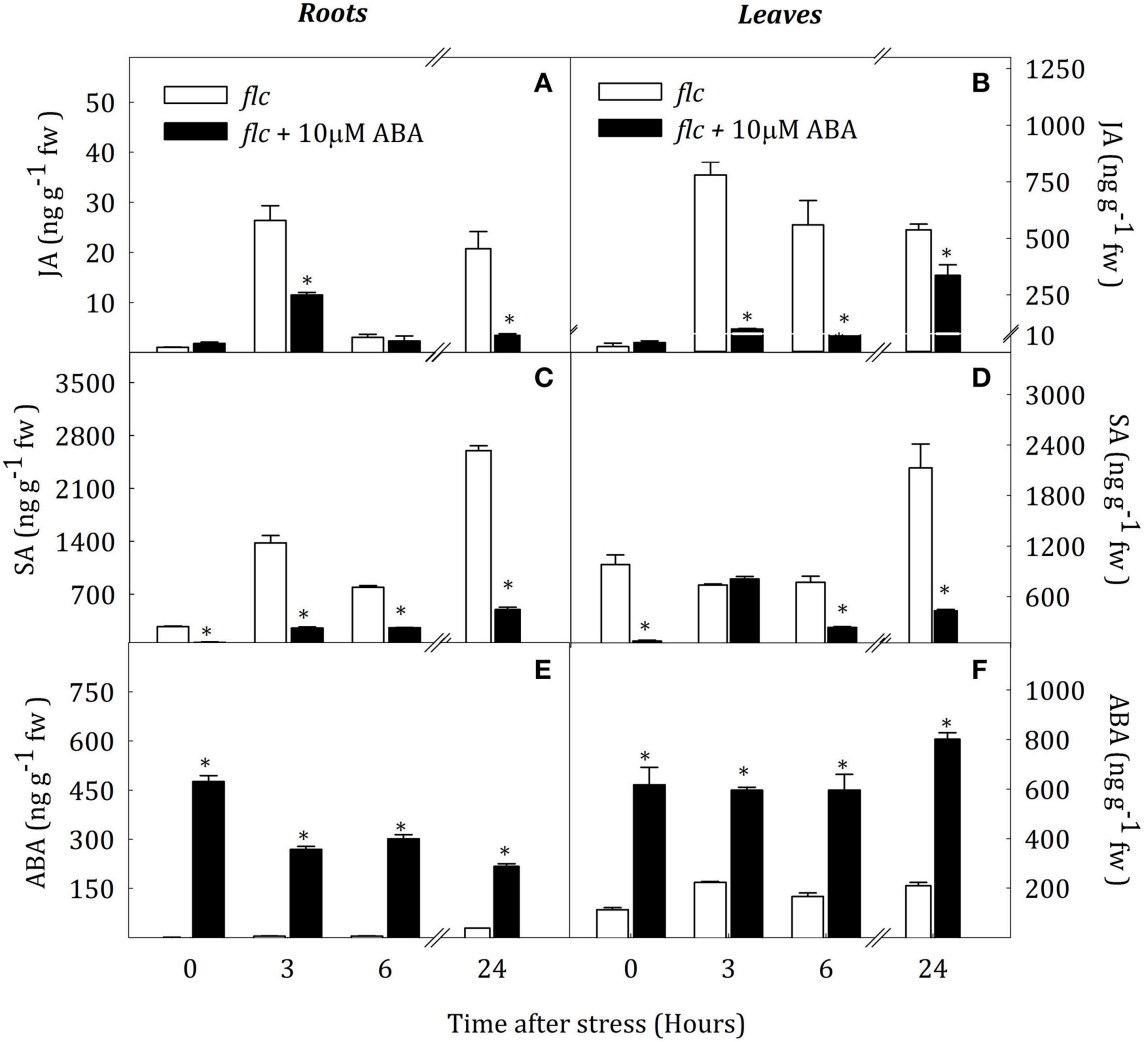
**JA (A,B), ABA (C,D) and SA (E,F) endogenous concentrations in leaves and roots of *Solanum lycopersicum* under control (*t* = 0) and water-stress conditions in *flc* seedlings without treatment (white bars) and *flc* seedlings and previously treated with 10 μM ABA (black bars)**. Data are mean values ± standard deviation of three independent determinations. Asterisks denote statistical difference with respect to WT at *p* ≤ 0.05.

To test the effect of ABA on SA and JA levels, *flc* mutants were treated with 10 μM ABA and thereafter exposed to water stress. Figure 9 shows that JA content remained on basal levels after ABA treatment in well-watered plants. On the contrary, SA levels decreased by the ABA treatment in roots and leaves (76.0 and 92.3% of control values, respectively). Under water deprivation, the pattern of JA accumulation was similar in roots of both groups of plants, although ABA-treated plants showed lower levels at 3 and 24 h of stress (56.5 and 83.4% with respect levels in non-treated plants, respectively). In contrast, ABA treatment reduced leaf JA levels during water stress. Non-treated plants showed a drastic increased of JA content after the onset of stress whereas ABA-treated plants only showed a slight accumulation at 3 and 24 h of stress, with values 87.2 and 37.4% lower than in non-treated plants (Figure 9B). ABA treatment reduced SA concentration in roots and leaves throughout the experimental period, except in leaves after 3 h of stress (Figures 9C,D).

### Hormonal profiles in a *NahG* line under water stress conditions

**Figure 10:**
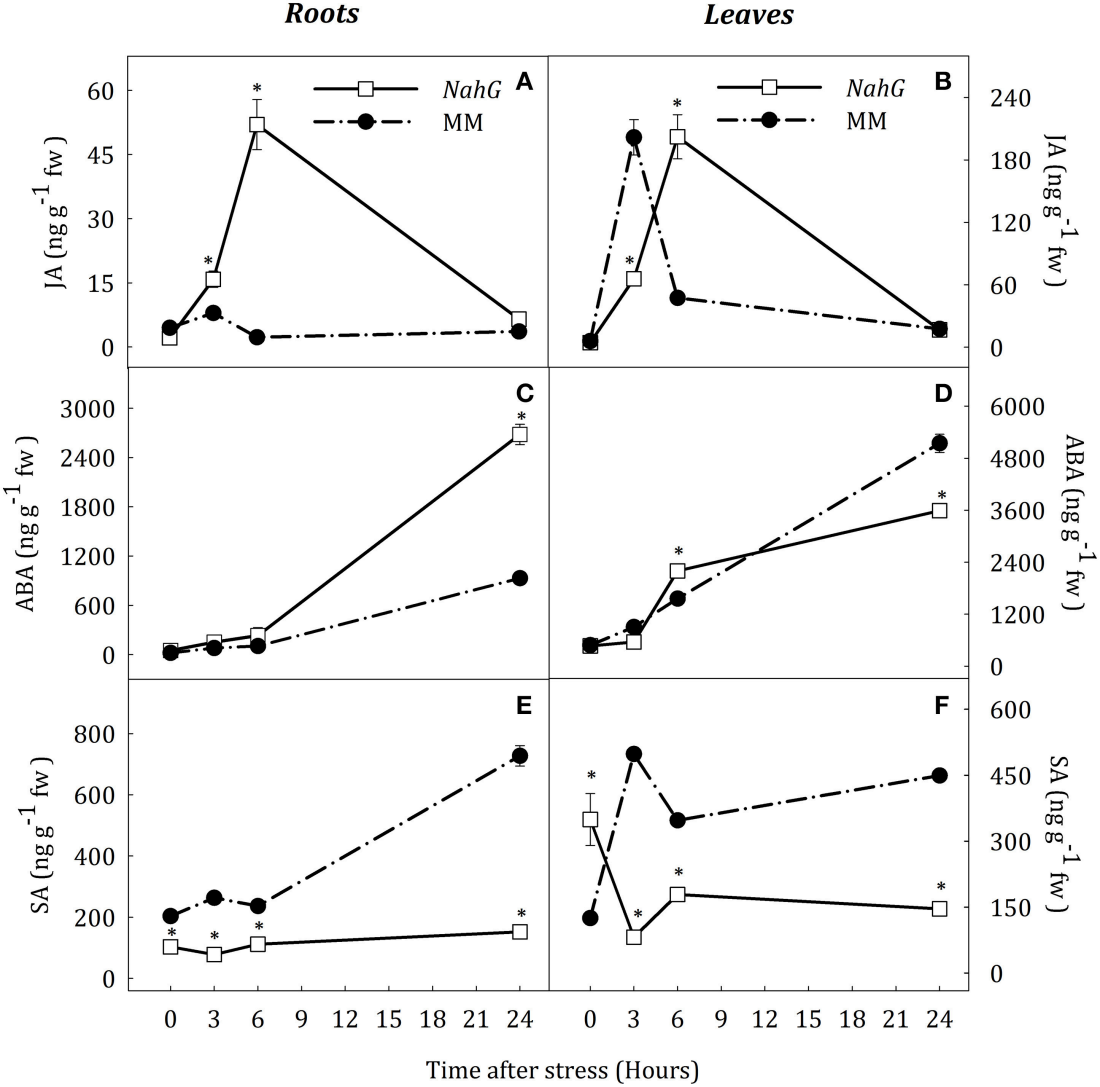
**JA (A,B), ABA (C,D) and SA (E,F) endogenous concentrations in leaves and roots of *Solanum lycopersicum* [WT (MM, black circles) and *NahG* line (white square)] under control (*t* = 0) and water-stress conditions**. Data are mean values ± standard deviation of three independent determinations. Asterisks denote statistical difference with respect to WT at *p* ≤ 0.05.

Based on previous results obtained with *spr2* and *flc* mutants, a transgenic *NahG* tomato line (cv. Moneymaker, MM) was used to evaluate the effect of SA deficiency on JA and ABA accumulation during water deprivation. Hormone profiles in MM plants under water deprivation were similar to those in AC and CSL plants (Figure 10). After 3 h of stress, MM plants showed a transient peak of JA and SA and also a progressive increase of ABA in both organs (Figure 10). In contrast, the pattern of JA and ABA accumulation in the *NahG* line was slightly different to that in WT. In roots of stressed *NahG* plants, JA content was higher than in MM (2.0- and 23.2-fold increase at 3 and 6 h, respectively; Figure 10A). ABA levels were only different at the end of the experiment (2.8 fold increase with respect to MM, Figure 10C). As expected, SA remained at basal levels in *NahG* roots throughout the experiment (Figure 10E). The pattern of JA accumulation in leaves of stressed *NahG* plants was similar to that in roots (Figure 10B). The transient increase of JA levels in these plants occurred after 6 h of stress whereas in MM plants this was observed at 3 h of stress. ABA accumulation in *NahG* leaves progressively increased after 6 h of stress, (not at 3 h as in MM), and the ABA levels in the transgenic plants were 30.2% lower than in MM at the end of experiment. Under stress, SA concentration in *NahG* leaves was lower than in MM (Figure 10) although basal levels at time 0 were higher in the transgenic line. Therefore, the analysis of the *NahG* line clearly suggests a crosstalk between JA and SA under water stress situations.

## Discussion

Increasing number of recent reports in the literature point to the importance of JA on drought signaling (Brossa et al., 2011; Daszkowska-Golec and Szarejko, 2013; De Ollas et al., 2013, 2015); however crosstalk with other hormones remains unclear. A further layer of complexity arises from antagonistic and synergistic interactions between hormones and their tissue-specific biosynthesis in relation to signaling under water stress conditions (Seo and Koshiba, 2002; Kazan and Manners, 2008). The present work indicates that SA, along with JA and ABA, is involved in signaling water stress responses. Moreover, our data demonstrate that the expression of key genes for the biosynthesis of these three hormones is induced by drought and that regulation of JA and ABA levels differs in roots and leaves.

Despite the involvement of ABA and JA in drought signaling (Fujita et al., 2011; De Ollas et al., 2013, 2015), some reports also indicate that SA improves tolerance to water stress (Singh and Usha, 2003). To test the relation among JA, ABA, and SA, tomato mutants deficient in JA and ABA (*spr2* and *flc*) were used. Unlike previous research in *Arabidopsis* (Brossa et al., 2011), our data showed that JA-deficiency in tomato neither increased water loss nor modified ΦPSII with respect to WT seedlings. Moreover, contrary to what was observed by Brossa et al. (2011), the low levels of JA in stressed *spr2* plants did not affect ABA accumulation in any of the organs analyzed.

To further analyze ABA biosynthesis during water stress, expression of key genes involved in this pathway was examined in *spr2* and *flc* mutants. *NCED* and *AAO* genes typically form multigene families showing different organ-specific patterns of expression and environmental responsiveness. In tomato, only *NCED1, NCED2*, and *NCED6* were known to be involved ABA biosynthesis (Taylor et al., 2005; Ntatsi et al., 2014). *NCED1* is the orthologous member of the *Arabidopsis* (*NCED3*) which strongly respond to water stress (Iuchi et al., 2001) although other genes of this family also respond to osmotic and salinity stress. Therefore, when one gene is inactivated, another family member may take over its function. The low levels of *NCED1* transcripts in *spr2* roots are consistent with previous studies showing that JA is required for its up-regulation (De Ollas et al., 2013, 2015; Du et al., 2014) under stress. However, despite the evident role of *NCED1* as key enzyme in ABA biosynthesis under water stress, up-regulation of *NCED2* in *spr2* plants together with the high levels of endogenous ABA found in this mutant suggest a possible compensative mechanism for ABA biosynthesis in this genotype. In contrast, the transient increase of JA in *flc* mutant was quite similar to that in AC plants though levels of *NCED1* transcripts were lower than in AC whereas *NCED2* was not up-regulated. This could be explained, at least in part, because *NCED1* expression might be negatively regulated by any of the intermediates in the ABA biosynthesis pathway. In this case, it is possible that *flc* mutant accumulated xanthoxin or abscisic aldehyde under water stress, considering the loss of function of AAO enzyme. Thus, we can speculate that these metabolites negatively affect the *NCED1* expression.

Previous reports have shown that *AAO3* is the major *AAO* gene involved in ABA biosynthesis in *Arabidopsis* (Seo et al., 2000) whereas in tomato *AAO1, AAO2*, and *AAO3* could perform this function. Data presented herein demonstrate that *AAO2* and *AAO3* are also induced by water stress in tomato roots. These results partially disagree with those of Yesbergenova et al. (2005) as they only describe an increase of *AAO3* expression in roots; however, differences may be explained by the moderate stress applied in that study vs. the more severe drought condition imposed in our work. Additionally, it has been demonstrated that SA can increase *AAO* expression (Szepesi et al., 2009). In this context, up-regulation of *AAO2* and *AAO3* in *spr2* and *flc* mutants under water stress, concomitant with the increase of SA content, suggests that SA could be responsible for the positive regulation of ABA levels in tomato roots. The lack of a clear correlation between SA and ABA levels in *NahG* plants under water stress could be explained in terms of different strategies developed to compensate the SA deficiency.

Molecular and hormonal data obtained in roots and leaves of both mutants used indicate a complex, organ-specific regulation of JA and ABA pathways. The unexpected lack of induction of *OPR3* in *flc* and *notabilis* roots together with the high induction of *OPR3* and the decreased of JA levels in *flc* leaves treated with ABA, suggest that ABA is involved in *OPR3* expression under water stress and their function differ from roots to leaves. In concordance with this result, a recent study in *Arabidopsis* (Savchenko et al., 2014) described a cooperative function of OPDA and ABA to control stomatal closure in response to drought, noticing the relevance of *OPR3*. Furthermore, previous reports have demonstrated the role of 12-OPDA in drought resistance (Seki et al., 2007; Grebner et al., 2013). Similar relation between JA and ABA was observed when *NCED1* expression in both mutants was analyzed in roots and leaves. The lower induction of *NCED1* in roots seems to be related to JA levels. However, both JA and ABA deficiency resulted in *NCED1* overexpression in leaves, indicating that both hormones are able to reduce *NCED1* transcript accumulation. In this line, a recent work in tomato (Du et al., 2014) indicated that one NAC transcription factor activated by JA (denominated JA2), can induce *NCED1* expression and that the JA2-NCED1 transcriptional module might be also monitoring the endogenous ABA status. Full activation of the JA2-NCED1 module by dehydration requires a basal level of ABA. These results indicate a feedback between JA and ABA where *OPR3* and *NCED1* genes may have different role depending on the plant organ.

Previous studies revealed that SA treatment contributes to improve acclimation to salt stress in arabidopsis, wheat and tomato (Shakirova et al., 2003; Singh and Gautam, 2013) although contradictory results suggest a negative effect of this hormone (Borsani et al., 2001). The hormonal patterns obtained in this research showed that SA is involved in the early stages of drought response. Furthermore, expressions of *ICS* and *PAL1* under water stress demonstrate that SA biosynthesis is activated in all groups of plants. In addition, the high expression of *PAL1* in roots and leaves of *flc* mutants together with the decrease of SA levels in *flc* plants treated with exogenous ABA indicate that ABA may reduce SA levels by modulating *PAL1* expression. Previous reports in tomato, bean and soybean have described a partial repression of PAL activity in leaves by increased ABA levels under biotic stress (Ward et al., 1989; De Meyer et al., 1999; Audenaert et al., 2002). Data obtained in this work show that ABA negatively regulates SA levels in roots and leaves of tomato also under water stress conditions.

On the other hand, the transient accumulation of JA and SA as an early response to stress could be related to an adaptive process. These results are consistent with those obtained in tomato after infestation by *Bemisia tabaci* (Rodríguez-Álvarez et al., 2015). Moreover, the high JA content in *NahG* stressed roots with respect to MM and the high SA content in *spr2* stressed roots with respect to CSL, support the assumption that there is a coordinated action between both hormones and an antagonistic effect between JA and SA depending on levels of each hormone.

Overall, the results presented in this work indicate that water deficit induced a transient accumulation of JA and SA in roots and leaves during early events of signaling and that both hormones are involved in ABA biosynthesis. This study expands the evidence of a complex interaction between JA and ABA during water stress that modulates the levels of both hormones in roots and leaves. Finally, accumulation of SA suggests that this hormone can elicit plant-adaptive responses to drought. A future challenge is to uncover the key elements that connect the JA-SA crosstalk and how they work at the molecular level.

## Author contributions

ML and VM performed the experiments at the greenhouse and laboratory. JC performed analysis at the laboratory. AG supervised the research. All authors contributed to write the manuscript.

### Conflict of interest statement

The authors declare that the research was conducted in the absence of any commercial or financial relationships that could be construed as a potential conflict of interest.
