# Supplementary material for: Water Stress Responses of Tomato Mutants Impaired in Hormone Biosynthesis Reveal Abscisic Acid, Jasmonic Acid and Salicylic Acid Interactions
Source: Front Plant Sci. 2015 Nov 18;6:997. doi: 10.3389/fpls.2015.00997 (PMC4649032; doi:10.3389/fpls.2015.00997)
Supplement: Supplementary file 1 [file Table1.DOCX]

| **Gene** | **Primer sequences (5'-3')** | **Accession numbers** |
| --- | --- | --- |
| **ABA biosynthesis** | | |
| **NCED1** | Fwd-CTTATTTGGCTATCGCTGAACC  Rev -CCTCCAACTTCAAACTCATTGC | SGN-U577478 |
| **NCED2** | Fwd-TGGTTTTCATGGGACATTCATTAG  Rev-ATCTCCCTTCTCAACTCAACTCCCTATTCC | EU912387 |
| **AAO1**  **AAO2**  **AAO3** | Fwd-GTGGAGGTTGGAGGAGGGGTTGATGTT  Rev-TGTGGTACTTCCAGCCGTTAATCCA  Fwd-TCGATTGTTGTAGAGGTTGGAGGGATT  Rev-GATGTAGTGCTCCCTGCCGTCAAC  Fwd-TCAATCGTAGTAGAGGTTGGAGGGATTG  Rev-GCTTGATTCCGATGTAGTGCTTCCA | SGN-U582239  AF258809  AF258810 |
|  |  |  |
| **JA biosynthesis** | | |
| **AOC** | Fwd-GCCTCTGCTGCTCTTAGAACC  Rev-CGAAGATAAGCAGGGCTTCC | SGN-U562649 |
| **OPR3** | Fwd-CGAGCAGAGAGCGACGGCCG  Rev-GGCTTCTCAGTGGATGATATTGG | SGN-U576938 |
| **SA biosynthesis** | | |
| **ICS** | Fwd-TCCAGGCTGAAGATGATGAG  Rev-TTATTCCAACCGCAAATTCA | NM_001247865 |
| **PAL1** | Fwd-GTCAAGGCAGCTCAGAAGCT  Rev-GCAAGGAATTGAAGTTCTGAGC | M83314 |
|  | **housekeeping control** |  |
| **GAPDH** | Fwd-ACAACTTAACGGCAAATTGACTGG  Rev-TTACCCTCTGATTCCTCCTTGATTG | U97256 |

**Table S1.** Sequences of the primers used for real-time PCR. The genes analysed were 9-cis-epoxycarotenoid dioxygenase (*NCED1* and *NCED2*), ABA aldehyde oxidase (*AAO1, AAO2* and *AAO3*), allene oxide cyclase (*AOC),* 12- oxophytodienoate reductase 3 *(OPR3),* isochorismate synthase *(ICS)* and phenylalanine ammonia lyase *(PAL1).*
