# Supplementary material for: Water Stress Responses of Tomato Mutants Impaired in Hormone Biosynthesis Reveal Abscisic Acid, Jasmonic Acid and Salicylic Acid Interactions
Source: Front Plant Sci. 2015 Nov 18;6:997. doi: 10.3389/fpls.2015.00997 (PMC4649032; doi:10.3389/fpls.2015.00997)
Supplement: Supplementary file 2 [file Image1.PDF]

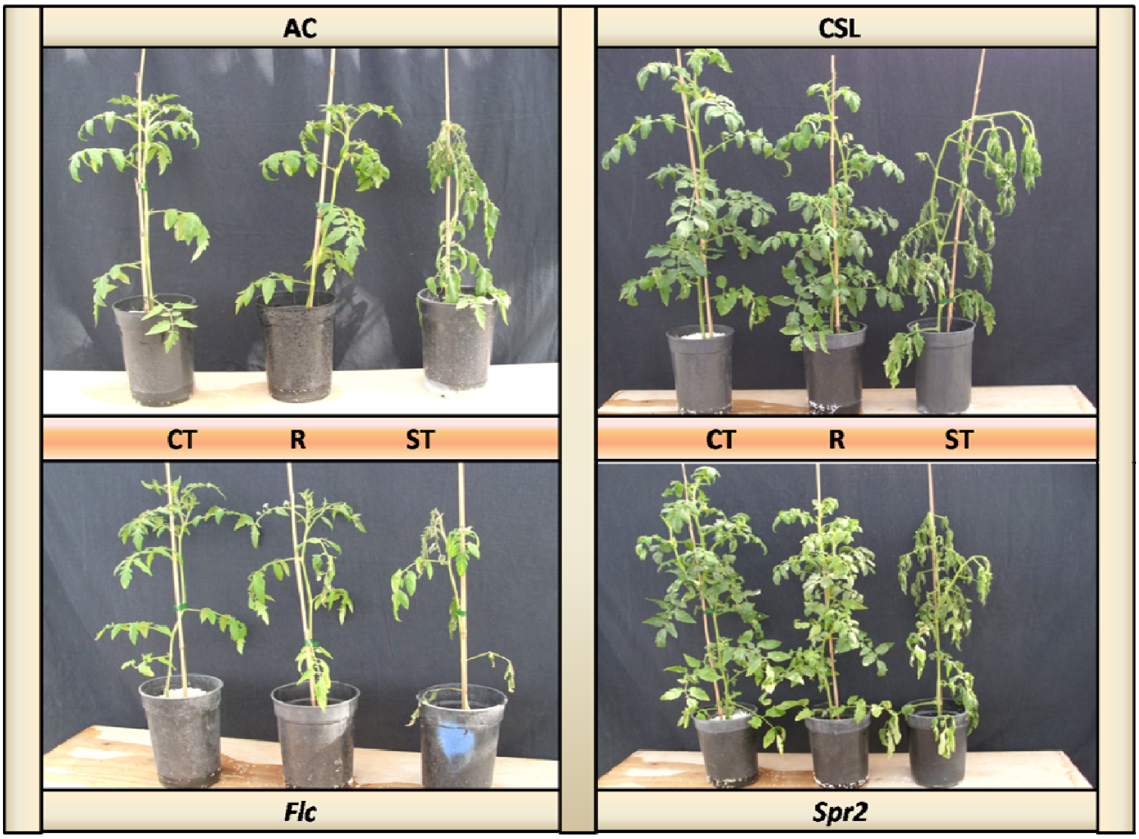

Figure S1. *Solanum lycopersicum* WT (CSL and AC) and mutants *(spr2* and *fl*c). (CT) control well-watered plants, (R) Plants stressed for 24 h and subsequently rehydrated, (ST) Plants stressed for 24 h.
