# Supplementary material for: Water Stress Responses of Tomato Mutants Impaired in Hormone Biosynthesis Reveal Abscisic Acid, Jasmonic Acid and Salicylic Acid Interactions
Source: Front Plant Sci. 2015 Nov 18;6:997. doi: 10.3389/fpls.2015.00997 (PMC4649032; doi:10.3389/fpls.2015.00997)
Supplement: Supplementary file 3 [file Image2.PDF]

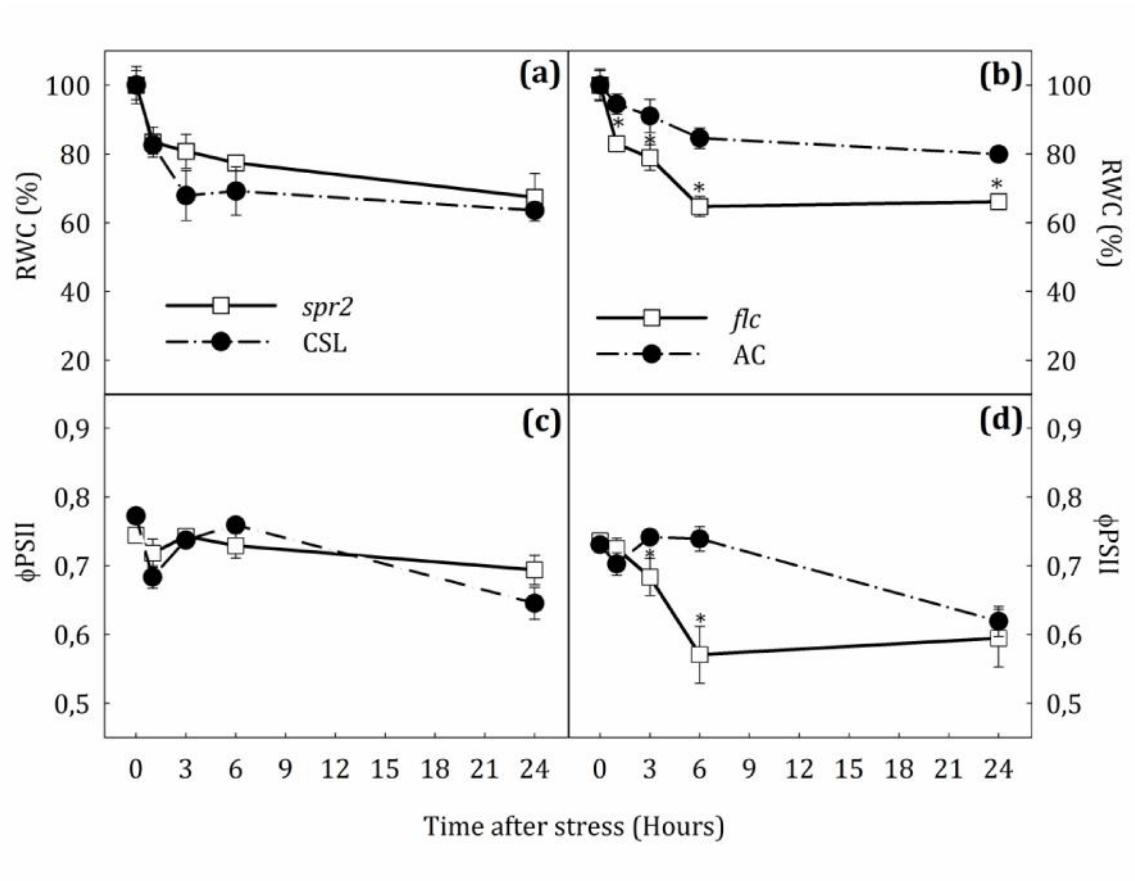

Figure S2. Relative water content (RWC) (a, b) and ΦPSII (c, d) in leaves of *Solanum lycopersicum*, [WT (CSL and AC, black circles) and mutants (*spr2* and *flc*, white square)] under control (t=0) and water-stress conditions. Data are mean values ± standard deviation of three independent determinations. Asterisks denote statistical difference with respect to WT at $p \leq 0.05$.
