# Supplementary material for: Water Stress Responses of Tomato Mutants Impaired in Hormone Biosynthesis Reveal Abscisic Acid, Jasmonic Acid and Salicylic Acid Interactions
Source: Front Plant Sci. 2015 Nov 18;6:997. doi: 10.3389/fpls.2015.00997 (PMC4649032; doi:10.3389/fpls.2015.00997)
Supplement: Supplementary file 4 [file Image3.PDF]

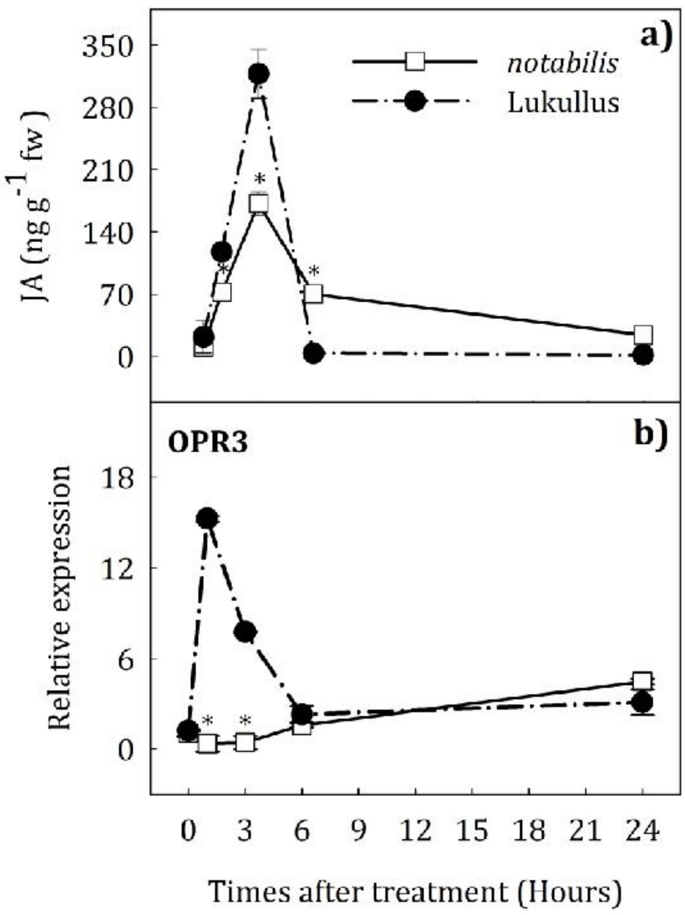

Figure S3. JA endogenous concentrations (a) and relative expression of *OPR3* (b) in roots of *Solanum lycopersicum* (WT Lukullus, black circles) and ABA-mutant (*notabilis*, white square) under control (t=0) and water-stress conditions. Data are mean values ± standard deviation of three independent determinations. Asterisks denote statistical difference with respect to WT at $p \leq 0.05$.
